# The Horse as a Producer of Antitoxins1Read before the Thirty-second Annual Meeting of the U. S. Veterinary Medical Association.

**Published:** 1896-01

**Authors:** Olof Schwarzkopf

**Affiliations:** Chicago, Ill.


### THE HORSE AS A PRODUCER OF ANTITOXINS.[1]

BY OLOF SCHWARZKOPF, V.M.D.,
CHICAGO, ILL.

AT a time when we hear so much of the coming "horseless age," when the praise is sung of the "silent steed," and the horseless carriage parades our streets, even the calm among us are joyous to see the horse once more in a new field of utility. Of the many uses to which the horse has been put by man for thousands of years none has ever been loftier than its recent employment as a producer of antitoxins for some of the most dreaded of human diseases.

It will be known to you that the preparation of antitoxins is based upon the discoveries of Behring, who proved "that the blood-serum of an animal rendered artificially immune against certain infectious diseases injected into another animal will protect it against such disease or even cure it after infection." The weak point of this new theory is our lack of understanding of the nature of "immunity," inasmuch as there are several theories, notably those of Pasteur, Metschnikoff, Buchner, Behring, Roux, and others. Each opposes the other, none fully explains its phenomena. We know, however, by empirical observation that certain animals or classes of animals are protected by Nature, are "naturally immune" against particular diseases which are fatal to others. We also know that a previous sickness of certain diseases of men and animals, such as scarlet fever, smallpox, yellow fever, influenza in horses, and many others, usually produces an "acquired immunity," at least for a

[1] Read before the Thirty-second Annual Meeting of the U. S. Veterinary Medical Association.

reasonable length of time, against another attack of the same disease. Moreover we know that we can render animals "artificially immune" against some infectious diseases by protective inoculation. Lately we seem to have reached the point when "curative immunity" can be produced in time to counteract the infection and ward off the fatal toxic effects of certain germ-diseases by the application of antitoxins. It is true that this new therapeutic applies only to a few diseases, diphtheria, tetanus, and a few forms of septicæmia, such as puerperal fever in women but it is no conjecture to conclude that others will be added in proportion as our knowledge of the pathogenic germ-life becomes clearer and our laboratory methods improve in practicability and certainty of results.

The production of the various antitoxins is preceded by the preparation of their toxins in the bacteriological laboratory. This has been already described in our professional journals, and only for a better understanding of this paper I wish to recall to you that the virulent bacilli of diphtheria and of tetanus and the streptococcus of septic diseases are secured from patients suffering from these diseases, and cultivated on bouillon in large flasks in an incubator for several weeks, when they are filtered through a Chamberland filter, whereby the germ remains outside the filter, the filtrate being the original fluid substrate charged with the toxin. The whole process is by no means simple, but calls for tedious work, painstaking accuracy, and patience, and in spite of all this may bring disappointment and unexpected difficulties, which must be overcome before further steps can be taken and success attained.

Having thus prepared the toxins, the animal for inoculation is selected, and it has been found that the horse is preferable to any other animal. He is most susceptible to these toxins, most tractable in the almost painless operations of remittent injections; he yields the largest amount of blood-serum from one bleeding, and most of all produces a therapeutic serum of the strongest immunizing value. The horses selected at our college for this purpose are healthy, strong, and large animals, of good disposition, between the ages of six and eight years, and they make a favorable impression upon those timid visitors who yet entertain doubts as to the value of serum-therapeutics.

What interests us most, as veterinarians, is the effect which the injections of the different toxins have upon the organism of the horse. There are certain symptoms, such as the rise of temperature, which are common to all toxins; otherwise, however, they are so widely different that it is well to give a separate description of each.

The inoculation with diphtheria-toxin has been lately described by Dr. Gill, of New York, and I shall, therefore, only briefly mention our own method. As the best places for injection we select the thorax and flanks, the proximity to the extremities being avoided on account of subsequent œdematous swellings. A piece of skin of the size of a dollar is shaved, washed, and disinfected with a solution of bichloride of mercury. The toxin is then drawn into a sterilized syringe of the capacity of 10 to 50 c.c., and slowly injected subcutaneously. We commence with a small injection of 2 c.c. of a weak toxin, and gradually increase during several weeks to 50 c.c.

The effect of diphtheria-toxin upon the system of the horse is mainly a rise in temperature of one and a half to three degrees, a quickened circulation, and accelerated respiration, but not in a very pronounced type. From one to four hours after the injection a certain uneasiness of the horse is sometimes observed, but grave symptoms, as described by others, have not been seen by us. I think that much depends upon the surroundings of the horse, and that a suitable diet and a comfortable and large box-stall prevent the horse from fully realizing the unpleasant feeling which no doubt he is undergoing. On the whole, however, the horses evidently enjoy the easy life with which they have been favored; they thrive, and become easily fattened.

Very different is the effect produced by the injection of tetanus-toxin. To the sceptic there could hardly be a more convincing proof of the scientific correctness of the elimination of toxins during the progress of some germ-diseases than to witness the effect of this particular toxin. After the injection of 2 c.c. of a weak tetanus-toxin, we observe within ten minutes very pronounced symptoms of ill-feeling. The rise of temperature follows almost immediately, the pulse becomes stringy, and abdominal breathing sets in. In about one hour the masseter became hardened and painful to touch, accompanied by a peculiar twitching of the muscles of the nose. In two hours and a half the temperature rose to 104.2°. Occasional grinding of the molar teeth was followed by outbreaks of great nervousness. At intervals the neck was thrown backward and upward and tetanic spasm of the muscles of the neck was noticed, resembling the symptoms observed in a horse that is trying to vomit. Later, light local spasms could be produced in any region by a simple touch. These symptoms lasted for about eight hours, when they gradually disappeared.

It is interesting to mark the effect of the subsequent injections of increasing quantity of toxin with a decreasing severity of symptoms. It is as if one could see the developing immunity, for the gradually increasing power of resistance of the horse against this toxin is very apparent. While our last injection was as large a dose as 25 c.c., beyond a rise in temperature to 104.4° we noticed none of the symptoms described as observed at the first injection.

There is great likelihood, however, that all horses do not react with equal gravity at the first injection. We inoculated experimentally an old and hardened horse with 20 c.c. of toxin, and anticipated very grave symptoms at first. For about an hour, however, the old horse stood motionless, exhibiting no reaction beyond a sudden rise in temperature of 2°. Then he showed gradually uneasiness, with occasional spasm of the neck and a peculiar arching of the back. Within two hours and a half the temperature rose to 104.2°, the horse became extremely sensitive to touch, and pressure at the point of injection in the shoulder produced spasm of the muscles in that region. After several hours, being suddenly approached by a stableman, he fell backward, and died within fifteen minutes under tetanic convulsions.

Symptoms still different are produced by the injection of streptococcus-toxin. The main effect is an elevation of temperature of one to two or more degrees, which extends over the second or even the third day. The other symptoms become visible within three to four hours after injection, and are not very marked but none the less severe. The horse stands rather quiet, the head bowed downward, paying no attention to its surroundings, noise, or food offered to him. He remains so for hours at a time, and he gives the impression of being a "pretty sick horse," as a visiting veterinarian expressed it the other day. After the fifth injection of this toxin the horse shows symptoms of colic-pains, looking at his flanks and biting them occasionally.

These are our observations up to date, and as far as I know the effects of the tetanus-toxin and streptococcus-toxin upon the horse have not been published before. Inasmuch as these observations were conducted on a limited number of horses they are subject to modification, but on the whole they will be found to be correct. The symptoms, as described, will greatly depend upon the preparation of the toxins, the germs producing different toxicity according to the soil on which they are cultivated. It is a matter of bacteriological experience to select a material of such chemical composition as will produce the highest toxicity, and much depends upon the skill of the bacteriologist. I think that some of the toxins if properly prepared may be profitably used for demonstrative purposes for students, as the effect is so marked and characteristic.

After the horses have been inoculated with these toxins for a sufficient length of time to endure large doses, and after having fully recovered from the effect of the last injection, a sample of blood is drawn, and the serum separated under aseptic precautions, and injected into experimental animals which have previously been infected If by this experiment the serum proves to contain sufficient antitoxic properties, large quantities of blood are drawn for the preparation of antitoxin for therapeutic purposes.

As to the application of the tetanus-toxin and streptococcus-toxin in veterinary practice, very few, if any, trials have yet been made, as these new therapeutic agents are of so very recent date. We treated one horse, which was beyond hopes of recovery, with a 10 c.c. dose of tetanus-antitoxin merely to satisfy the owner, who wanted us to try anything to save the horse. Although this was the entire quantity we had on hand, and too small a dose, the owner informed us the morning after the injection that there was a noticeable improvement in the condition of the horse, but he died the same night. I report this negative result in order to dispel extreme expectation. As reports from human hospitals indicate, the early application of tetanus-antitoxin, if accompanied by proper surgical treatment of the wound from which the infection started, is bound to result in a cure. With the frequency and gravity of tetanus in the horse we certainly look toward this new therapeutic agent with great hopes, and it remains to be seen whether its success in veterinary practice will be equal to that in human medicine.

Whatever may be the future of serum-therapeutics, I should warn you against too much enthusiasm. Let us cautiously try these new remedies, if they are such, and not give a judgment off-hand, as has already been done by some. Success will alternate with failure, and the supporters and enemies will view it from an opposite standpoint. As far as veterinary science is concerned it is certain to play an important part in the development of serum-therapeutics, and to us as members of this science it will add greater responsibilities and insure greater opportunities.

